# Detection of pelagic habitat hotspots for skipjack tuna in the Gulf of Bone-Flores Sea, southwestern Coral Triangle tuna, Indonesia

**DOI:** 10.1371/journal.pone.0185601

**Published:** 2017-10-02

**Authors:** Mukti Zainuddin, Aisjah Farhum, Safruddin Safruddin, Muhammad Banda Selamat, Sudirman Sudirman, Nurjannah Nurdin, Mega Syamsuddin, Muhammad Ridwan, Sei-Ichi Saitoh

**Affiliations:** 1 Faculty of Marine Science and Fisheries, Hasanuddin University, Makassar, Indonesia; 2 Center for Region, Spatial Planning and Information, Hasanuddin University, Makassar, Indonesia; 3 Faculty of Fisheries and Marine Science, Padjadjaran University, Bandung, Indonesia; 4 Fisheries Agribusiness Department, National Agricultural Polytechnic, Pangkep, Indonesia; 5 Faculty of Fisheries Sciences, Hokkaido University, Hakodate, Japan; Havforskningsinstituttet, NORWAY

## Abstract

Using remote sensing of sea surface temperature (SST), sea surface height anomaly (SSHA) and chlorophyll-a (Chl-a) together with catch data, we investigated the detection and persistence of important pelagic habitat hotspots for skipjack tuna in the Gulf of Bone-Flores Sea, Indonesia. We analyzed the data for the period between the northwest and southeast monsoon 2007–2011. A pelagic hotspot index was constructed from a model of multi-spectrum satellite-based oceanographic data in relation to skipjack fishing performance. Results showed that skipjack catch per unit efforts (CPUEs) increased significantly in areas of highest pelagic hotspot indices. The distribution and dynamics of habitat hotspots were detected by the synoptic measurements of SST, SSHA and Chl-a ranging from 29.5° to 31.5°C, from 2.5 to 12.5 cm and from 0.15 to 0.35 mg m^-3^, respectively. Total area of hotspots consistently peaked in May. Validation of skipjack CPUE predicted by our model against observed data from 2012 was highly significant. The key pelagic habitat corresponded with the Chl-a front, which could be related to the areas of relatively high prey abundance (enhanced feeding opportunity) for skipjack. We found that the area and persistence of the potential skipjack habitat hotspots for the 5 years were clearly identified by the 0.2 mg m^-3^ Chl-a isopleth, suggesting that the Chl-a front provides a key oceanographic indicator for global understanding on skipjack tuna habitat hotspots in the western tropical Pacific Ocean, especially within Coral Triangle tuna.

## Introduction

Pelagic habitat hotspots, which are defined as areas of high biological activity where linkages occur between physical processes, primary production, secondary consumers and higher tropic level predators, play an important role in controlling distribution, migration and abundance for commercial and wide-roaming pelagic species in many different oceans [1,2,3]. The distinct oceanographic signatures in turn signify enhanced trophic interactions, physiological and foraging advantages, and thus provide high ecological and economic importance. Large pelagic fish as well as commercial fishing vessels recognize that prey organisms aggregate at ocean hotspots, which are mostly represented by ocean fronts, eddies and upwelling zones [2,3,4]. Thermal fronts are important congregating spots for many valuable pelagic species in Baja California-Bering Sea [5]. In the western Mediterranean, the spatial pattern of bluefin tuna school distributions was determined by the key oceanic habitats (i.e. fronts and eddies) [6]. Using multi-spectrum satellite images, hotspots for albacore tuna in the western North Pacific Ocean correspond with surface fronts and eddies [7,8]. Albacore forage habitat and migration routes are driven by the dynamic features of a pelagic hotspot namely a. Chl-a front known as the TZCF in the eastern and central Pacific Ocean [9]. Recent findings suggest that the frontal area, eddy field, and topographic features (seamount) are important habitat hotspots for pelagic species such as flying squid and tuna [10,11,12]. Therefore, detection of the ecologically significant pelagic habitats and their spatial persistence is critical for marine management strategies and identifying potential targets for conservation.

Skipjack tuna (*Katsuwonus pelamis*) is one of the most valuable species in the world in terms of catch weight [13]. It is a main target of a high-value commercial fishery in the tropical region, accounting for more than one half (approximately 58%) of the global tuna catch [13]. Between 2005–2014, the fish contributed 47% of Indonesia total tuna catch [14]. Hence, understanding of the species optimal habitats is central to evaluating fishing strategies and sustainable pelagic fisheries resources within Coral Triangle area.

The potential habitat for this species is within the warm surface layers of tropical and subtropical oceans [15,16]. Several oceanographic studies have found that skipjack tuna migration, distribution and abundance are linked with oceanic fronts and eddies [17,18,19] and are strongly influenced by ambient temperature and dissolved oxygen concentration [20,21]. In the western North Pacific Ocean, Sea Surface temperature (SST) and surface Chl-a were found to be more important variables of skipjack [22]. SST is one of the key oceanographic parameters to study skipjack tuna habitat in tropical region [23]. The occurrence of pelagic hotspots (salinity front and convergence zone) identified with 29°C SST isotherm provides a reasonable proxy to detect the region of highest skipjack CPUEs in western Pacific Ocean [24].

There are many studies that assess skipjack tuna habitat around the world using various methods. Skipjack forage habitats in the Pacific Ocean have been predicted based on Spatial Ecosystem and Populations Dynamics Model [15,23,25]. To characterize the spatial pattern of skipjack tuna habitat in the western North Pacific, generalized additive models (GAMs) and GIS techniques have been combined [22]. Using boast regression trees, the potential impact of climate change on skipjack tuna habitat in the Intra Americas Sea (IAS) has been discussed [26]. In the eastern central Atlantic and western Indian Oceans, favorable feeding habitats for skipjack have been investigated using a single ecological niche model [27]. The recent findings show that skipjack tuna habitats in different El Nino events can be identified based on the optimal model of Habitat Suitability Index (HSI) [28]. Most of the previous analyses of the preferred skipjack tuna habitat use statistical, ecological and spatial population dynamics models. The spatial persistence of the fish’s habitat has rarely been presented. In the present paper, we develop a model to explore not only habitat hotspots for skipjack tuna but also their persistence using multi-spectrum satellite images and high resolution of fishing performance data. This paper also highlights the important association between chlorophyll front and the skipjack habitats in the western Equatorial Pacific (southwestern Coral Triangle tuna).

The Coral Triangle, which primarily encompasses the seas of Indonesia, Papua New Guinea and the Philippines is a known tuna (skipjack, yellowfin and bigeye) nursery and migratory path, producing about 46% of all tuna catches in Western and Central Pacific Ocean [29,30]. The Gulf of Bone—Flores Sea is an important coral reef area located in the southwestern Coral Triangle where many commercial tuna fisheries conduct fishing operations. Our preliminary study estimated the MSY (Maximum Sustainable Yield) for this study area is 49,709 tonnes per year, indicating the great potential skipjack fishing ground. Statistical data (2007–2013) from Agency For Marine and Fisheries Affairs, South Sulawesi Province indicate that trend of skipjack catch tends to increase during the period between northwest and southeast monsoon. During this period, surface temperature gradually decrease while Chl-a tends to be high, providing high biological productivity [31] which are in turn correlates with high catches of skipjack [32].

Several investigations have assessed skipjack tuna habitat in the western tropical Pacific Ocean especially northern waters of Papua (Indonesia) and Papua New Guinea [15,23,24]. Those areas are predominantly located in the eastern area of Coral Triangle tuna. However, there is a critical gap of information about skipjack tuna distribution in the opposite area (southwestern Coral Triangle tuna, particularly in the Gulf of Bone- Flores Sea). This area is one of the most potentially great tuna fishing grounds in Indonesia waters [31,32]. The aims of the present paper are to detect a spatial pattern of pelagic habitat hotspots for skipjack tuna and to map out their persistence in the southwestern Coral Triangle tuna using remotely sensed satellite and catch data.

## Data and methods

### Study area

**Fig 1:**
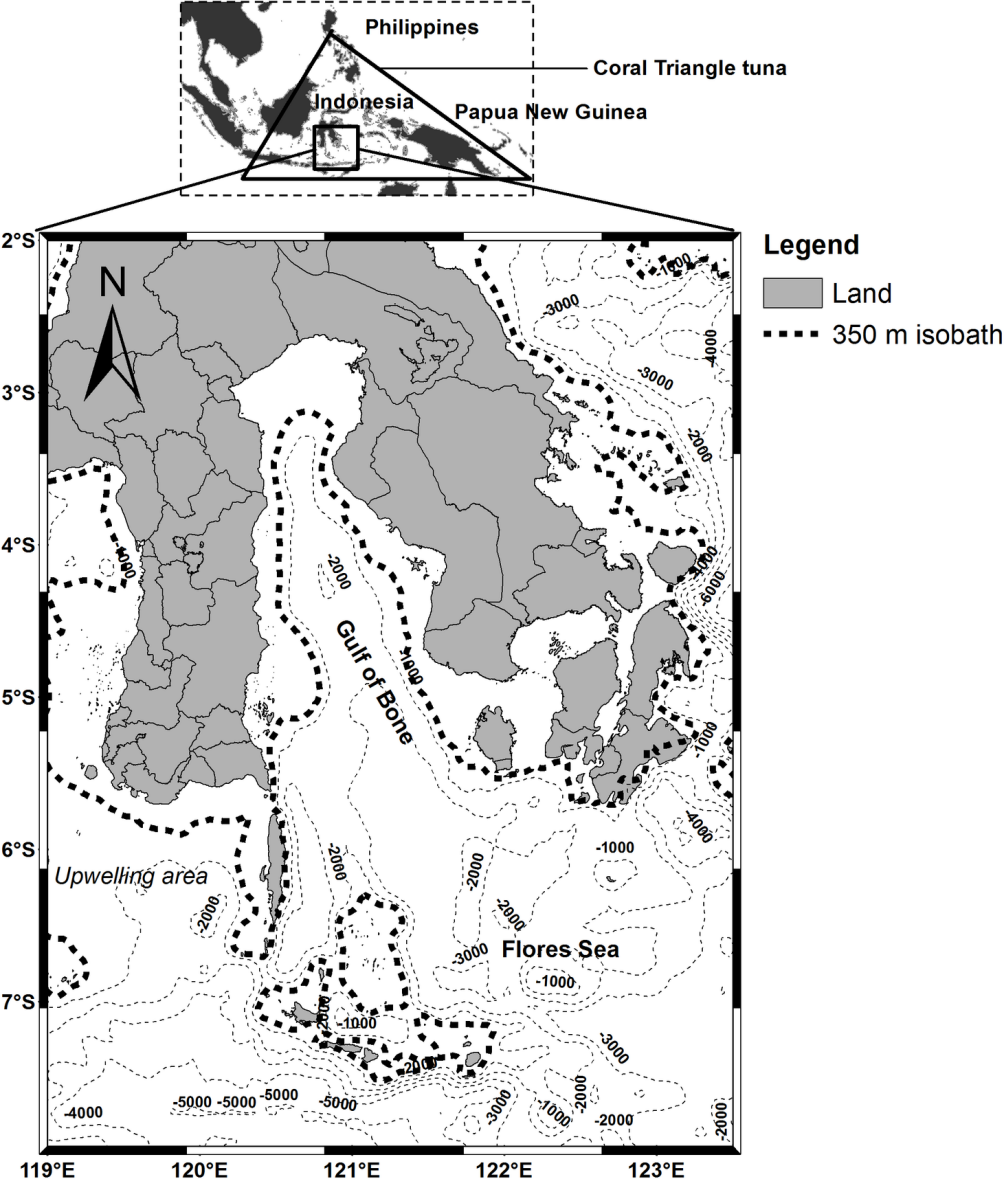
A location map of the southwestern Coral Triangle tuna showing the major oceanographic and bathymetric features. The broken lines correspond to the spatial position of 350 m isobath (shelfbreak).

The Coral Triangle tuna, so named because of its distinct triangular shape, contains nearly 5.7 km^2^ of coral reefs and spans parts of six countries: Indonesia, Malaysia, Papua New Guinea, the Philippines, Solomon Islands, and Timor-Leste [29]. The area of interest, the Gulf of Bone- Flores Sea located in the southwestern Coral Triangle tuna is one of the most biologically productive skipjack fishing grounds (Fig 1). In addition, the study area is also known as one of the main pathways of the Indonesian throughflow (ITF) and is strongly influenced by a tropical monsoon type of climate, resulting from the Asia-Australian monsoon wind systems, which change the wind direction with the seasons, i.e. southeast monsoon and northwest monsoon [31]. The interaction between the ITF and the Asian monsoon affects the specific current circulation system, Ekman mass and heat transport, tidal mixing, wind induced upwelling and down-welling systems and environmental variability of sea surface temperature (SST) and surface Chl-a concentration (hereafter Chl-a) [31,33,34]. Dynamics of the biophysical oceanographic structures in this area, results in a highly productive pelagic habitat hotspot, which serves as a forage ground for various commercially and ecologically important pelagic species including tuna [32,35].

### Pole and line fishery data

The pole and line fishery in the study area, which extends from 118.5°E to 122.5°E longitude and 2°S-8°S latitude, captures the skipjack tuna mostly between the northwest and southeast monsoon (January-June). The fishery catch data were collected from pole and line fishing logbooks provided by the Fish Landing Bases in Luwu and Sinjai, South Sulawesi, and the and Kolaka Districts and Incorporated Company of Indonesian Government, PT. Perikanan Samudra at Kendari, Southeast Sulawesi in the period between northwest and southeast monsoon 2007–2011. The fishery data comprised daily geo-referenced fishing positions (latitude and longitude), catch in number of skipjack and effort (fishing set), from which catch per unit effort (CPUE) was determined in number of fish per fishing set, further compiled into monthly resolution datasets. To validate our model, the catch data were also collected from as many as 140 sampling fishing positions from scientific pole and line fishing surveys in the study area during the same period in 2012.

### Satellite remote sensing data

The physical and biological environmental data used to describe the oceanographic condition around the fishing locations are surface Chl-a concentration and sea surface temperature (SST). Terra/ MODIS (Moderate Resolution Imaging Spectroradiometer) level 3 standard mapped images (SMI) data were used to estimate sea surface Chl-a concentration and SST at all pole and line fishing ground locations. NASA distributes the level 3 binary data with HDF (*Hierarchical Data Format*) format. We obtained these data from NASA GSFC’s Distributed Active Archive Center (DAAC) (http://oceancolor.gsfc.nasa.gov/). For this study, we used Global Area Coverage (GAC), monthly mean MODIS images with a spatial resolution of about 4 x 4 km for the study period during 2007–2011 (Table 1).

**Table 1:** Summary of oceanographic parameters used for developing habitat hotspot models for skipjack tuna in the Gulf of Bone-Flores Sea, southwestern Coral Triangle tuna, Indonesia.

Oceanographic variables	Abbreviation	Temporal Resolution	Spatial Footprint	Data Source
**Sea surface temperature**	SST	Monthly	4 km	Terra/MODIS
**Surface chlorophyll-a**	Chl-a	Monthly	4 km	Terra/MODIS
**Sea surface height anomaly**	SSHA	Daily	25 km	AVISO

In the present study, we used SSHA data distributed by AVISO (the Archiving, Validation and Interpretation of Satellite Oceanographic data). The SSHA data were global images with 0.25° spatial resolution in both longitude and latitude. Due to the different spatial and temporal resolutions with SST and Chl-a, the SSHA data were resampled into the spatial footprint (4 km) and sampling interval (monthly) spatial resolutions and then subset to the study area. Monthly values of all satellite images (SST, Chl-a and SSHA) were extracted from each pixel corresponding to the location of fishing activities using spatial analyst of ArcGIS 10.3. The result was a full matrix of the skipjack tuna CPUE as well as the environmental variables. All satellite images were processed using IDL (Interactive Data Language) software package and had the same spatial and temporal resolutions prior to the model construction.

### Construction of pelagic habitat hotspot map

To detect the spatial pattern of the skipjack pelagic hotspots throughout study area, we constructed a model of fishery performance, which took into account both CPUE (index of fish abundance) and frequency of fishing effort (index of fish occurrence) in relation to the three oceanographic variables. This model was improved and developed from the albacore hotspot model [7] by adding a weighting factor, allowing the contribution of each variable on the pelagic hotspot index (PHI) was taken into account. In addition, we added SSHA variable into the model to address the relationship between skipjack tuna and mesoscale variability.

The habitat hotspot was determined using environmental probability indices, reflecting the high probability areas of finding skipjack tuna. Specifically, the PHI was computed based on total CPUE at a given interval of histogram divided by the maximum total CPUE from all class intervals of the three variables (SST, SSHA and Chl-a) (Eq 1), and fishing frequencies were also calculated with the same method (Eq 2). The variable which has the highest CPUE or fishing frequency (maximum value) was used a standard. Then, we calculated the average of probability indices from the interval ranges of all variables (Eq 3). The highest probability value in which the probability index is more than 0.75 (PHI > Quartile 3) indicated the pelagic habitat hotspots, showing the greatest probability areas of finding the fish. In contrast, the lowest probability denoted the least suitable locations for detecting skipjack tuna. Lastly, we combined the three satellite images to create a pelagic hotspot map for all interval ranges of the environmental conditions.

The CPUE data were then overlain on the map and the probability index of the joint environmental factors was extracted from each pixel corresponding to the fishing ground positions. The probability area was visualized using ArcGIS 10.3 Spatial Analyst software package. Then, based on the distribution of data points, we employed the piecewise regression technique to examine the relationship between total CPUE and the hotspot index around fishing locations. To evaluate the strength of the relationship, we used the correlation coefficient (r). Here this attempt focused on an analysis of the pelagic hotspots in the seasons of highest skipjack abundance from 2007–2011. For validation, we analyzed catch and the environmental data during the same period in 2012 using both spatial distributions of fishing data on the hotspot map and the correlation analysis. All the habitat hotspot images were mapped using spatial analyst toolbox in ArcGIS software package. The model used to calculate pelagic habitat hotspot index (PHI) as follows:

$$\mathrm{PI}\mathrm{cpue}=\frac{\sum \frac{{\mathrm{cpue}}_{\mathrm{ij}}}{{\mathrm{cpue}}_{i}\max}}{n}$$ (1)

$$\mathrm{PI}f=\frac{\sum \frac{F_{\mathrm{ij}}}{F_{i}\max}}{n}$$ (2)

$$\mathrm{PHI}=\frac{\left(\mathrm{PI}\mathrm{cpue}+\mathrm{PI}f\right)}{2}$$ (3)

Where PHI is the pelagic hotspot index; PIcpue is the mean probability index for skipjack based on the relationship between CPUE and the three oceanographic variables (SST, Chl-a, SSHA) for each histogram graph; PIf is the mean probability index based on the relationship between fishing frequency and the oceanographic variables for the histogram graphs; cpue_ij_ is the value of CPUE in relation to oceanographic variable-i for class interval-j; cpue_i_ max is the maximum value of CPUE among the oceanographic variables; F_ij_ is the value of fishing frequency in relation to oceanographic variable-i for class interval-j; F_i max_ is the maximum value of fishing frequency among the oceanographic variables; n is the total number of variables.

### Detection of persistent pelagic habitat hotspot

A persistent pelagic hotspot map was constructed based on the presence or absence of the strong environmental probability index (probability of more than 75%) in the study area. We built the persistent hotspot map by computing monthly mean composite hotspot images at the peak season between the northwest and southeast monsoon 2007–2011. The map consisted of value ranging from zero (0) to five (5). The highest value (5) indicated that the persistent hotspot at a certain spatial location took place during the period of five years. While, the lowest value (0) denoted that there was no persistent hotspot available at a given area during at least one year. Then, we overlaid the conspicuous environmental characteristics on the map to find a reliable proxy indicator for locating the persistent skipjack habitat hotspots.

## Results

### Temporal variation of catch data and environmental variables

**Fig 2:**
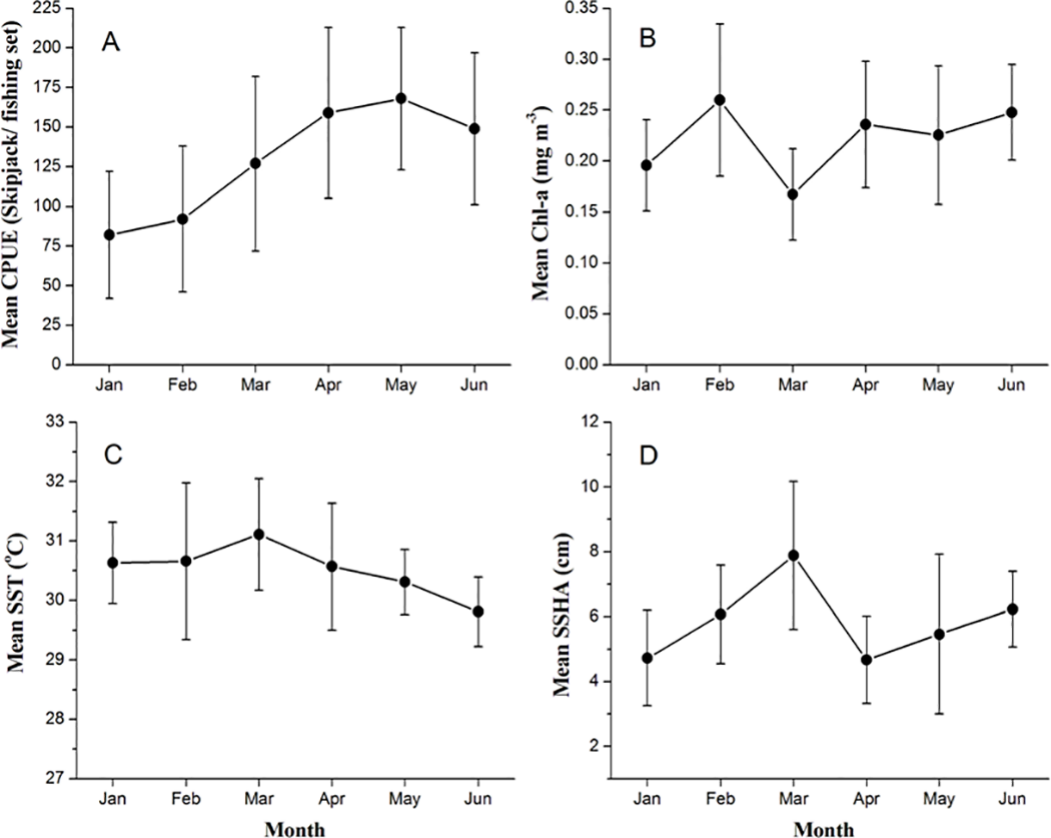
Temporal variability of (A) CPUE of skipjack fishery, (B) SST, (C) Chl-a concentration, and (D) SSHA, between northwest and southeast monsoon (January-June) 2007–2011.

During the period of April-June, skipjack CPUEs tended to be high and reached the peak in May (Fig 2A). Catch level in this month was about 170 fish/fishing set. The highest CPUEs occurred in areas of relatively high Chl-a and warmer SST ranging from 0.16 to 0.3 mg m^-3^ (0.22±0.068 mg m^-3^) (Fig 2B) and from 29.76 to 30.86°C (30.31±0.55°C) (Fig 2C), respectively. At the same time, the greatest skipjack catches were obtained in waters of positive SSHA ranging from 3.04 cm to 7.96 cm (5.50 ± 2.46 cm) (Fig 2D). During January-March, the catch rates (CPUEs) appeared to be lower than those of subsequent months. During that period, the fishing sets occupied the locations where surface temperature was relative high and Chl-a as well as SSHA fluctuated highly.

### Skipjack tuna in relation to environmental variables

**Fig 3:**
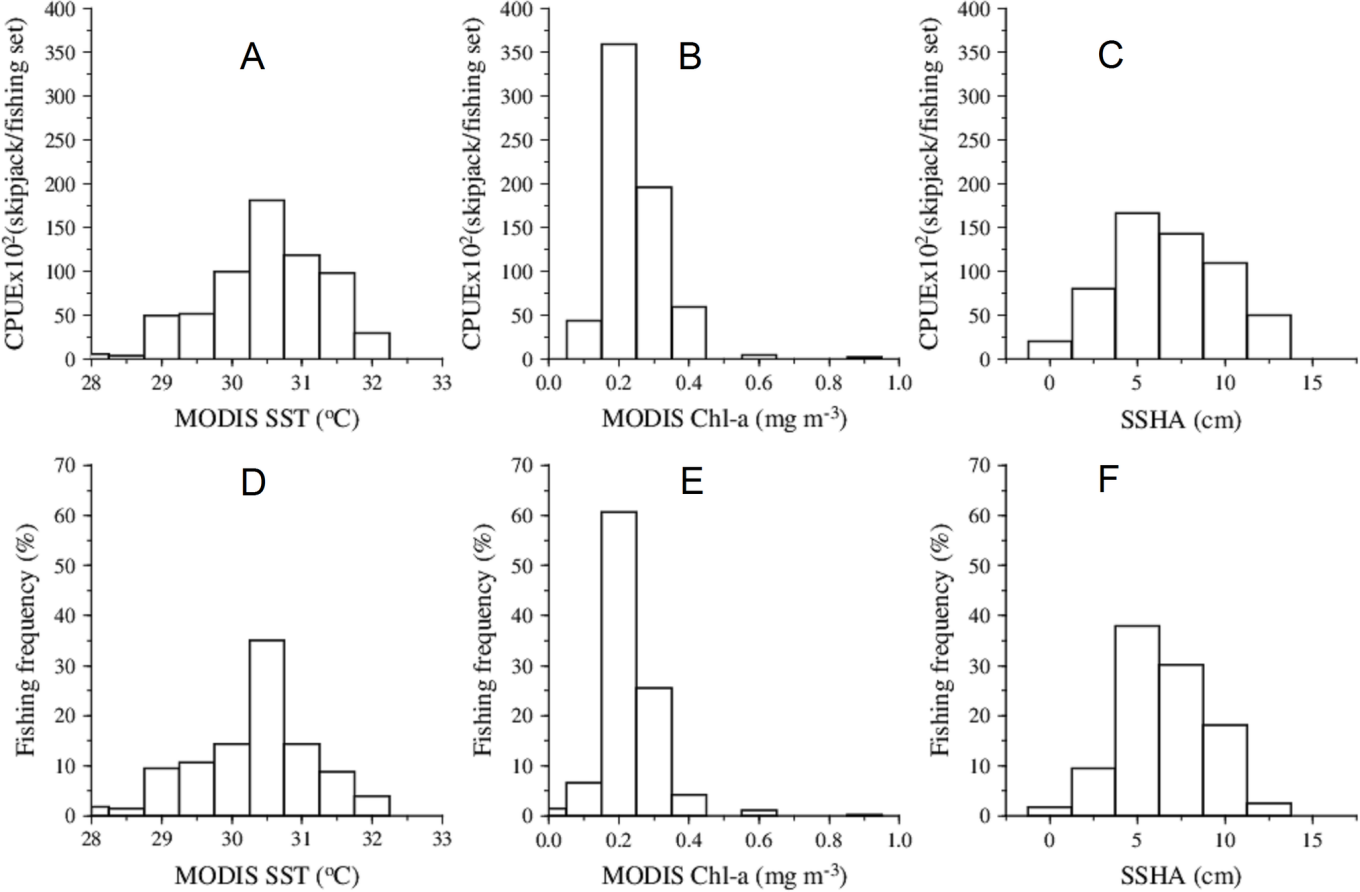
Total skipjack CPUE (skipjack/fishing set) in relation to MODIS SST (A), MODIS Chl-a (B), and SSHA (C) and fishing frequency in relation to SST *(*D), Chl-a (E) and SSHA (F) during January-June 2007–2011.

Satellite based oceanographic data in relation to skipjack tuna fishing performance indicated the specific ranges where the fish were most abundant (Fig 3). Total CPUEs in relation to SST showed that most of the catches were concentrated in areas where SST ranged from 29.75 to 31.25°C using histogram graph (Fig 3A). The similar trend was found in the relationship between the frequency of fishing set and SST (Fig 3D). Both histograms revealed that the preferred SST tended to center at 30.5°C, which reflected the highest probability of finding fish in term of SST. Total skipjack CPUEs in relation to Chl-a indicated that skipjack CPUEs were mainly found in areas where the environmental variable occurred mainly from 0.15 to 0.35 mg m^-3^ (Fig 3B). The relationship between skipjack fishing frequency and the surface Chl-a also showed a similar pattern (Fig 3E). The Chl-a preference for skipjack tuna mostly concentrated at 0.2 mg m^-3^. Whilst skipjack catches and fishing sets were derived in substantial number in waters where SSHA varied between 0 and 12.5 cm (Fig 3C). Both fishing performance reached an average at approximately 6 cm (Fig 3C–3F).

It is worth noting that Chl-a was the most important oceanographic variable to explain skipjack fishing performance. Specifically, we found that chlorophyll concentrations of about 0.2 mg m^-3^ was a good proxy for describing the highest total skipjack CPUEs (~54%) and fishing frequency (~60%) (Fig 3). Whilst, the value of SST 30.5°C was capable of exposing the catch rates of approximately 29% and frequency of the fishing set of about 35%. The optimum SSHA value of near 6 cm accounted for the skipjack CPUE and frequency of the fish occurrence were about 28% and 40%, respectively.

**Fig 4:**
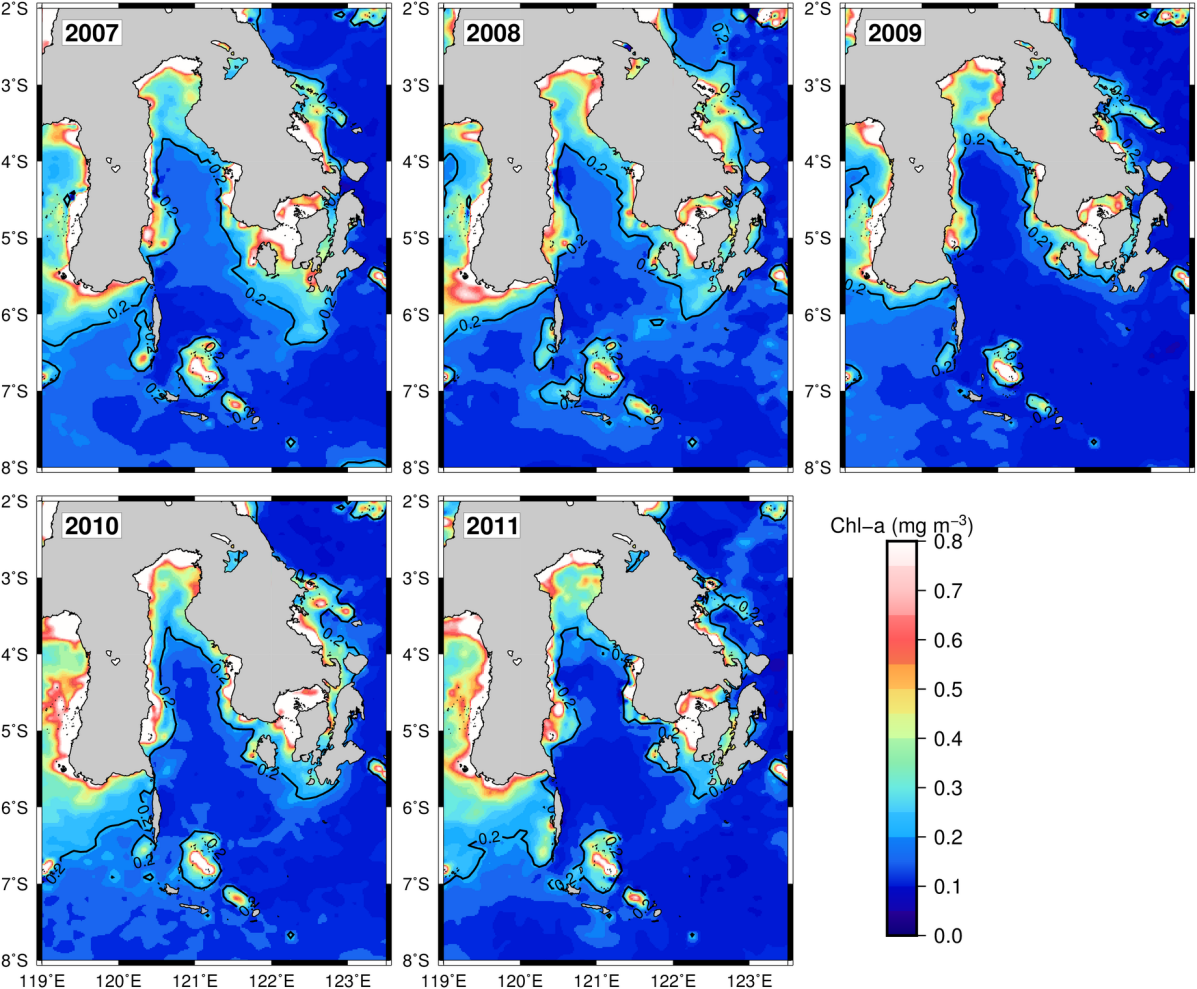
The spatial position of the Chl-a front measured by the 0.2 mg m^-3^ Chl-a concentration contour for May 2007–2011 estimated from MODIS ocean color data. The solid lines correspond to the Chl-a front along the study area.

The associated highest catches with the Chl-a front formed every year during 2007–2011 (Fig 4). The chlorophyll front consistently occurred in a specific location within the study area. Thus, the potential habitat was constantly available for the fishery every year (2007–2011) based on the environmental indicator. For the SST variable, the dynamics and position of the optimum range varied widely both in longitude and latitude and sometimes disappeared over the Flores Sea for instance in May 2008 (S1 Fig). Likewise, from the SSHA images, the spatial position of the most suitable range was widely distributed (S2 Fig).

### Pelagic habitat hotspot map for skipjack tuna

**Fig 5:**
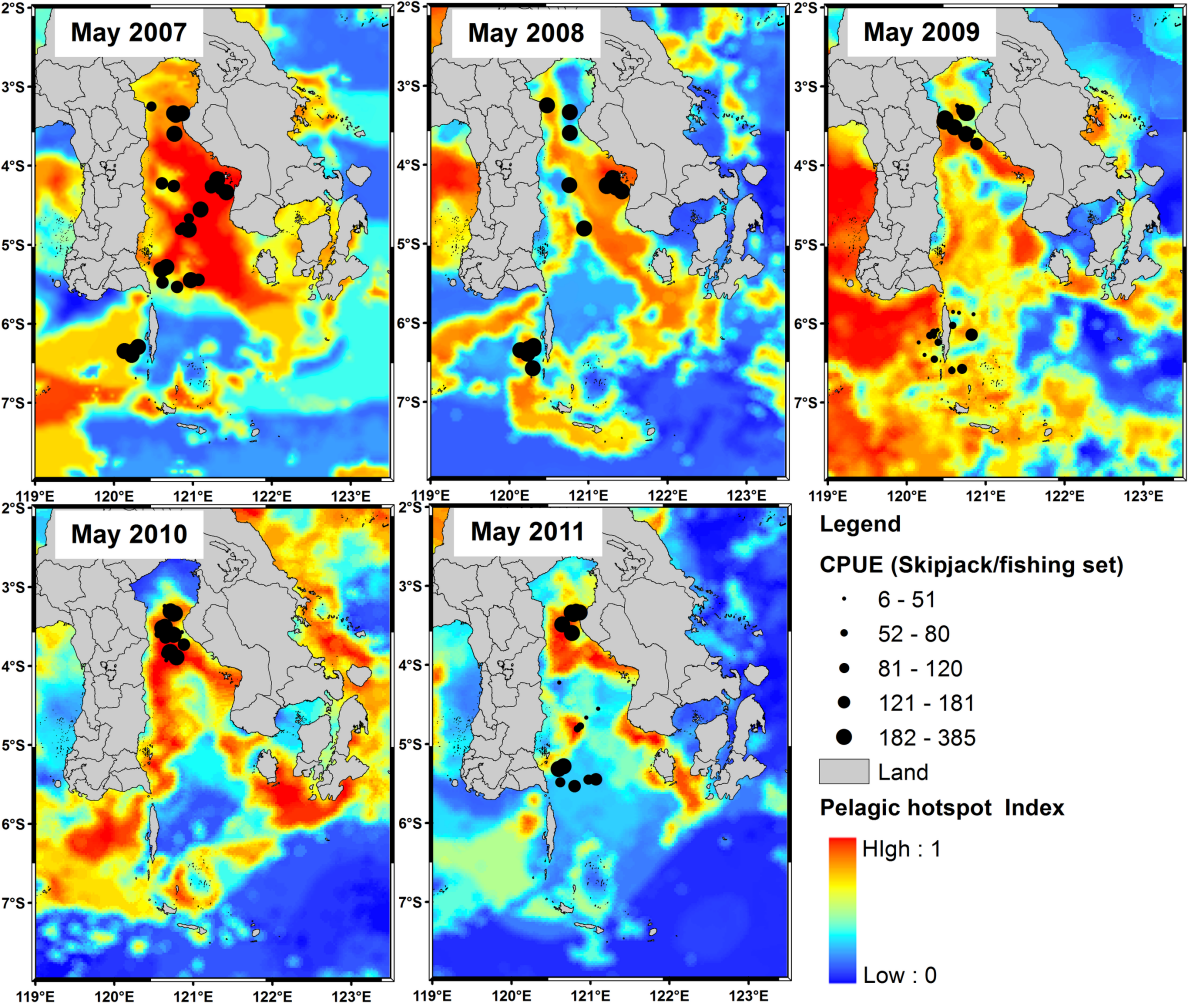
The spatial distribution of skipjack CPUE (skipjack/fishing set) from the pole and line fishery shown as dots for May 2007–2011 overlain on pelagic hotspot maps generated from a model of satellite images (Chl-a, SST and SSHA) in relation to fishing performance.

Areas of potentially suitable habitat hotspots for skipjack tuna strongly developed in May and covered the waters of approximately 8971 km^2^ on average (Fig 5). Mean PHI throughout the study area in the peak season was about 0.60. In contrast, the lowest pelagic habitat hotspot index occurred in January and occupied the areas of 2317 km^2^ with mean hotspot index of 0.41.

**Fig 6:**
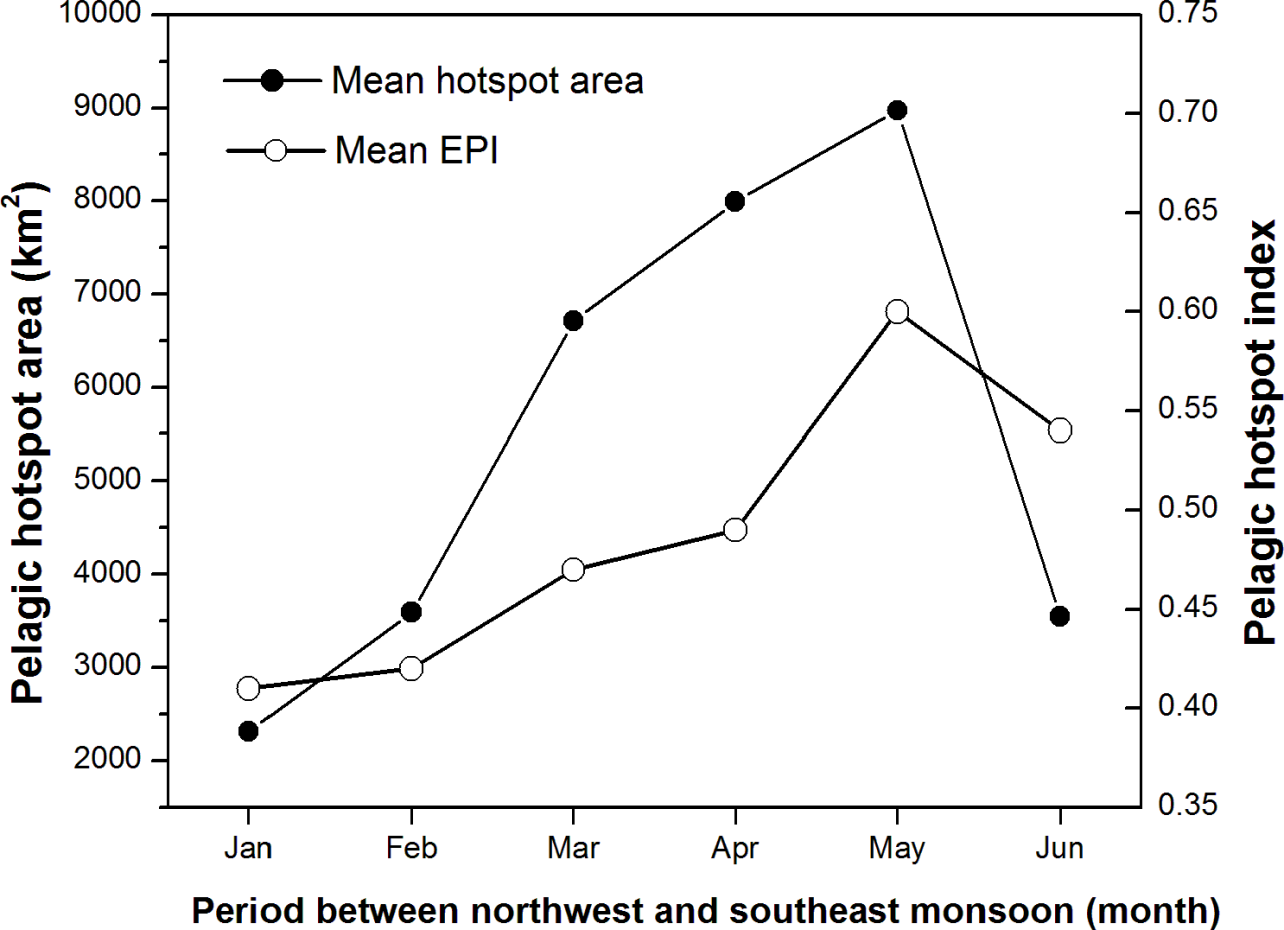
Monthly mean temporal variability of pelagic hotspot area (km^2^) and pelagic hotspot index between northwest and southeast monsoon 2007–2011.

During five years period, the spatial dynamics and intensity of habitat hotspots appeared to change significantly (Fig 6). However, it is important to note that a Chl-a of 0.2 mg m^-3^ isopleth was a good indicator for detecting spatial distribution patterns of the pelagic hotspots for all years (Figs 4 and 6). In 2007, the most suitable habitat strongly formed within the Gulf of Bone and was associated with the skipjack fishery distribution. The pelagic habitats were predicted to be in the eastern Bone Gulf and western Flores Sea in the subsequent year. We found that skipjack catches mainly concentrated in the hotspot area. In 2009, the predicted hotspots were mostly found in the western Flores Sea, whilst the skipjack tuna seemed to be captured in the hotspot areas of the northern Bone Gulf. Then, in the following year 2010, the pelagic habitat hotspots developed with the chlorophyll front and were associated closely with the fishery locations in the northern Bone Gulf. For the year of 2011, the habitat hotspots were well formed again in the northern area but with narrower both latitudinal and longitudinal bands and they matched generally with fishing data. In all years, it seems that the potential habitat also had a good association with the shelf-break formation (at the depth of about 350 m).

**Fig 7:**
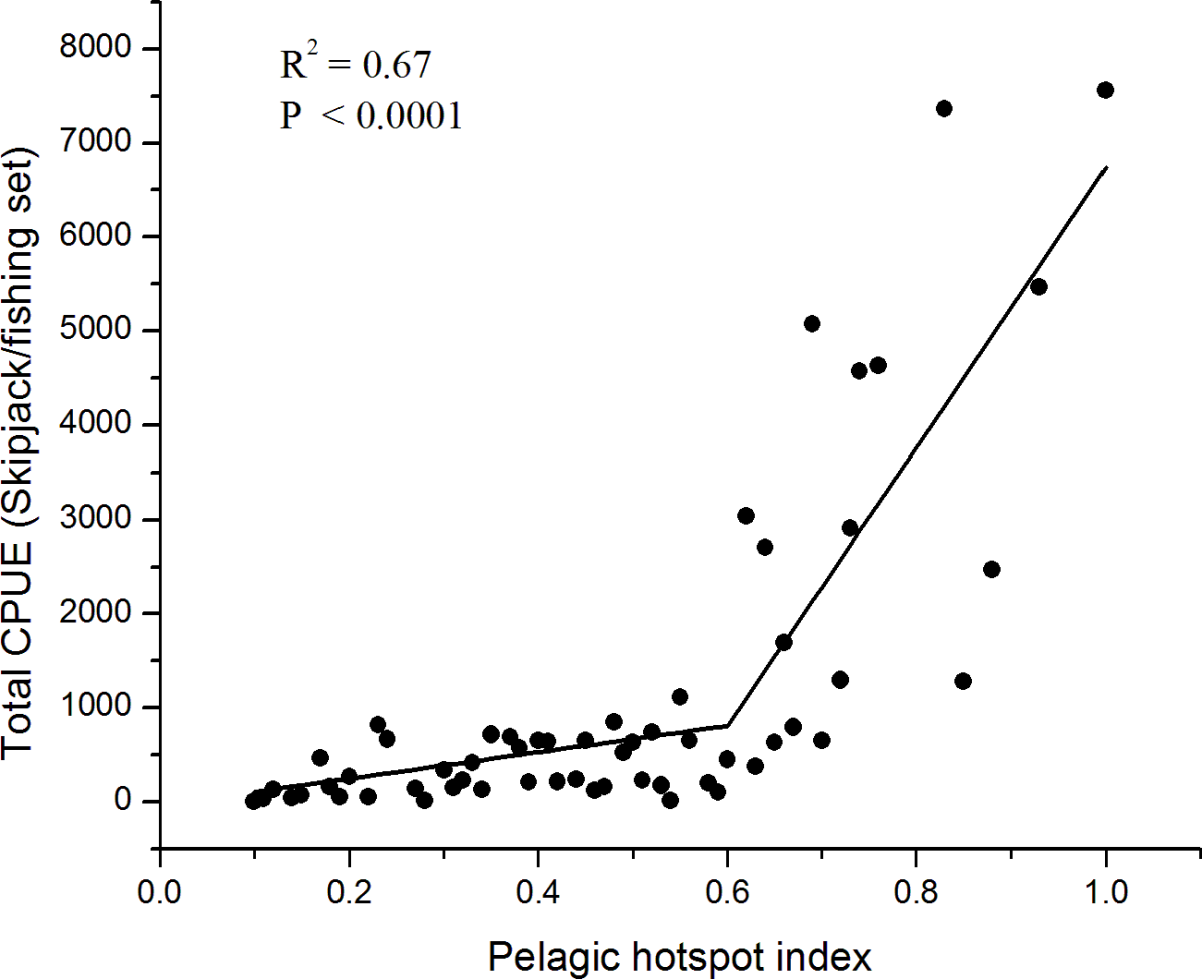
The relationship between total skipjack CPUE and PHI in the southwestern Coral Triangle tuna using piecewise linear regression.

The datasets for the period of northwest-southwest monsoon 2007–2011 showed that the total CPUEs significantly increased with the increasing probability values of joining environmental variables (R^2^ = 0.67, P<0.0001) (Fig 7). The increasing CPUEs were substantially found when the pelagic hotspot indices were more than 60%. The first equation of the regression lines was *Y = b*_*0*_*+b*_*1*_*X*_*1*_, when *X*_*1*_ ≤ 0.6 (X = 0.6 indicates the point where the slope change), and the second equation was *Y = (b*_*0*_*- 60b*_*2*_*)+(b*_*0*_*+b*_*1*_*)X*_*1*_ when *X*_*1*_>0.6. Therefore, we suggested that the PHI of joint oceanographic variables provided a reasonable proxy for predicting pelagic hotspots for skipjack tuna.

### Prediction and validation of skipjack CPUE

**Fig 8:**
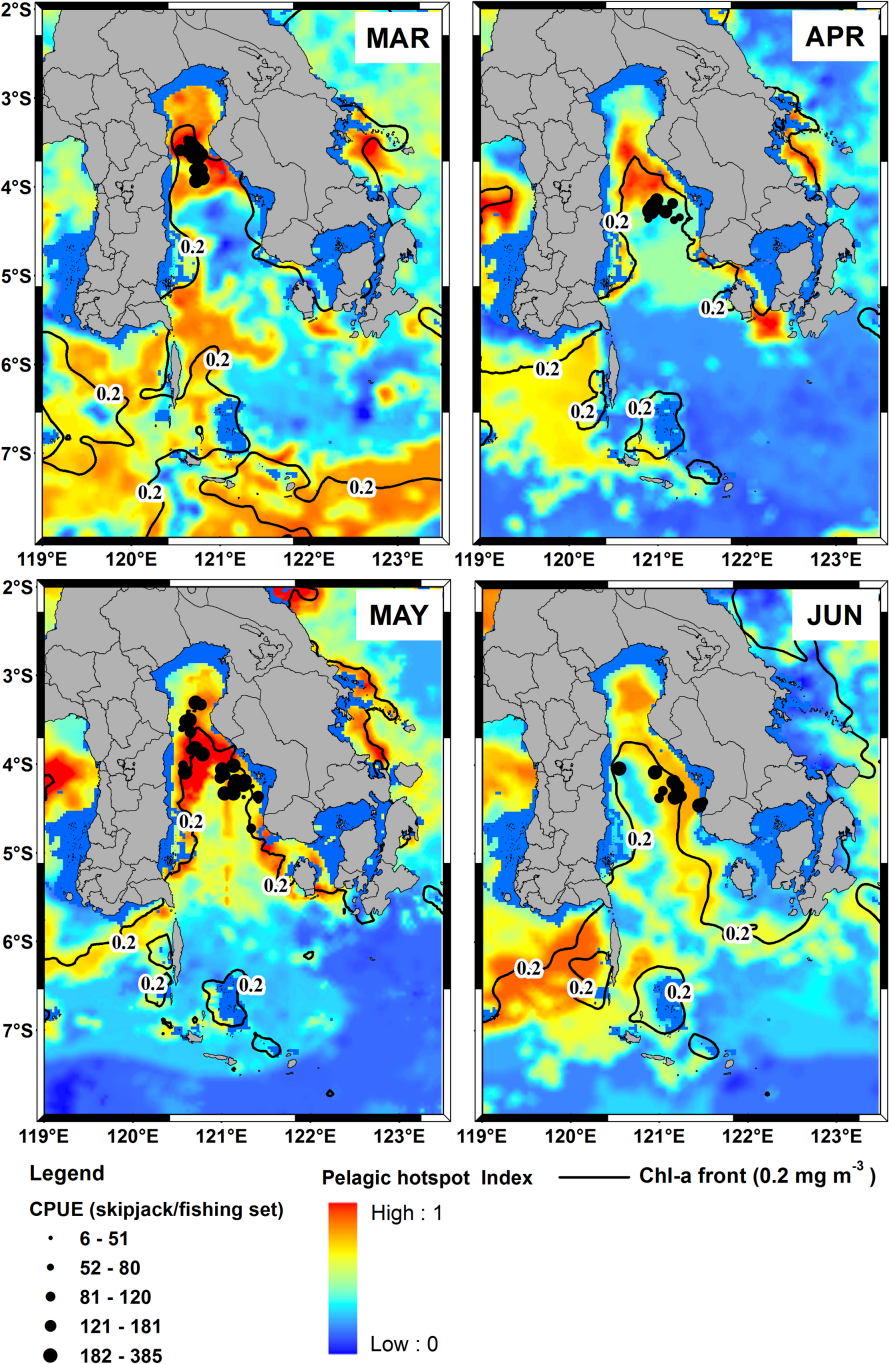
The spatial distribution of skipjack CPUE (skipjack/fishing set) shown as dots from March to June 2012 superimposed on the pelagic habitat hotspot map and Chl-a front. There is no fishing data during January-February 2012.

**Fig 9:**
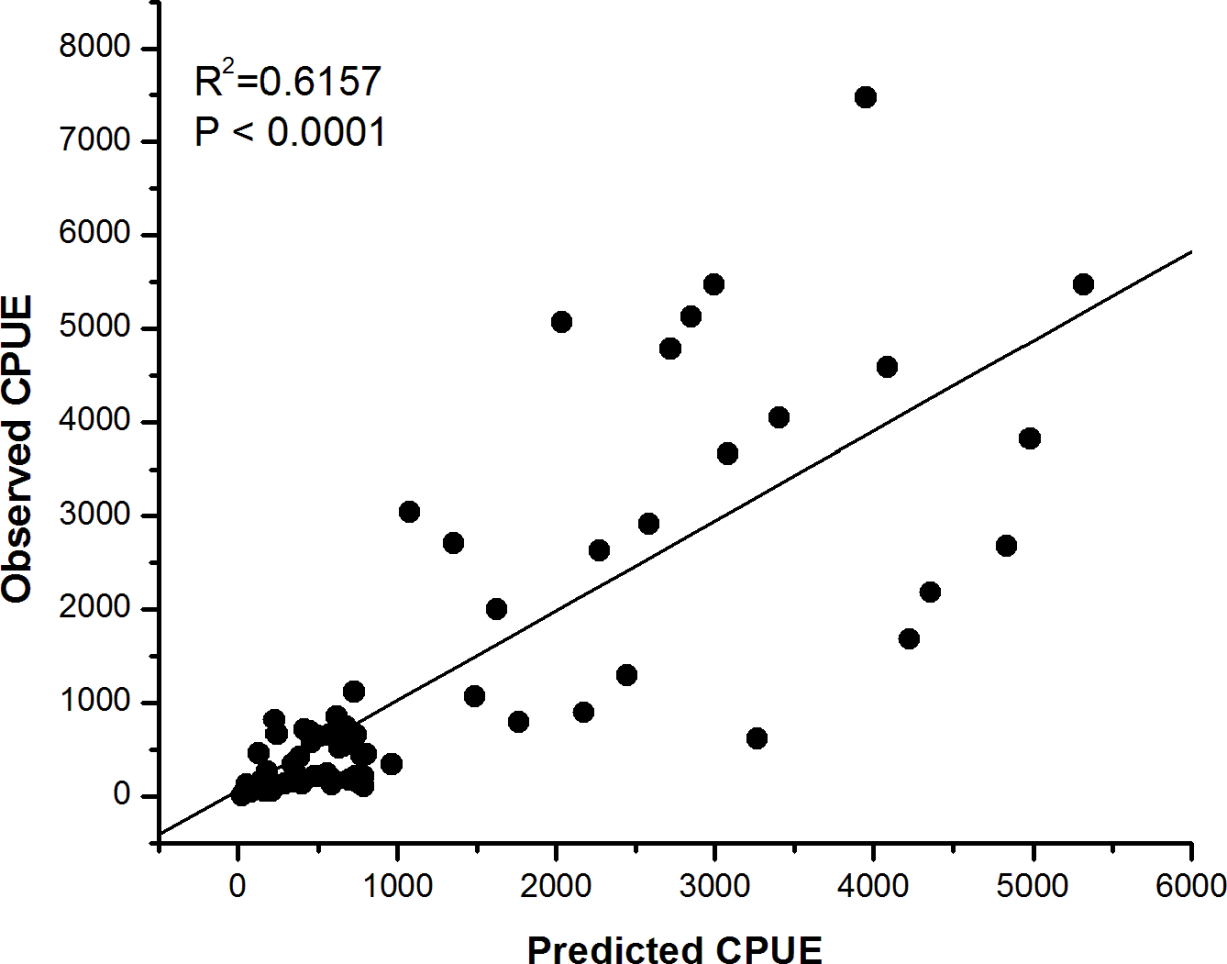
A scatter plot of pooled monthly observed against predicted skipjack CPUE values calculated from the pelagic hotspot index (PHI) (P < 0.0001, R^2^ = 0.6157).

For the spatial model validation, Fig 8 showed that the spatial distribution of the fishing data during March-June 2012 mostly occurred on predicted habitat hotspots (PHI > 0.75). The important skipjack habitats located the areas of 120.5–121.5°E longitude and 3.25–4.5°S latitude. It is interesting to see that the mean geographical position of the habitat hotspot was highly consistent with the Chl-a front position along the study area. Using pelagic habitat hotspot index as a predictor for skipjack CPUE response, we found that the correlation of predicted skipjack CPUEs against the observed was highly significant (P < 0.0001, R^2^ = 0.6157) (Fig 9). It inferred that during the period between the northwest-southwest monsoon, the pelagic hotspot model was significantly predicted skipjack CPUEs.

### Persistence of habitat hotspots for skipjack tuna

**Fig 10:**
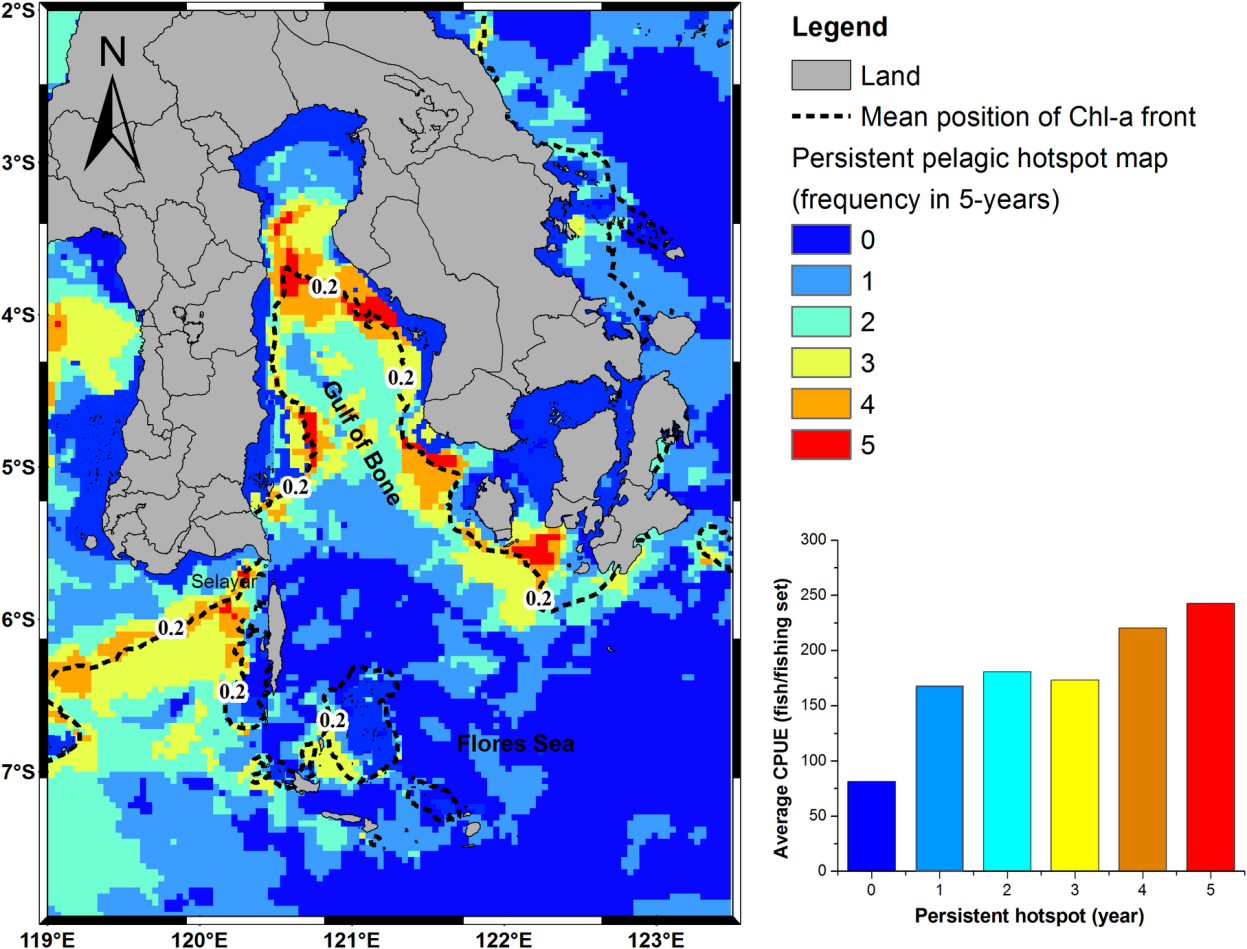
Spatial distribution of persistent pelagic habitat hotspots for skipjack tuna in the peak season May 2007–2011 (frequency/ 5 years) in the southwestern Coral Triangle tuna, Indonesia (left) and the graphical relationship between average CPUE and persistent habitat hotspots (right).

During the period of 5 years (May 2007–2011), the persistent habitat hotspots were found only in May and June (Table 2). The greatest persistent area occurred in May and covered approximately 1.21% of the grid cells in the southwestern Coral Triangle tuna for 5 years (Fig 10 and Table 2). These cells were all concentrated along the specific areas from the western Flores Sea, surrounding the Gulf of Bone to eastern Flores Sea. Nevertheless, our analysis indicated that more than 95% of the study area did not have persistent habitat hotspots throughout the study area. However, all persistent habitat hotspot formations associated consistently with the Chl-a front indicated by 0.2 mg m^-3^. Skipjack CPUE tended to increase at the most persistent habitat (Fig 10).

**Table 2:** Persistence of habitat hotspot location for skipjack tuna, in number of pixel per year, in the Gulf of Bone-Flores Sea, southwestern Coral Triangle tuna, Indonesia.

Month \ Year	2007	2008	2009	2010	2011
**January**	3846	894	78	4	0
**February**	4540	966	89	5	0
**March**	3923	2614	170	7	0
**April**	3082	2738	910	43	2
**May**	3386	1874	1233	735	193
**June**	3166	2403	1004	387	119

## Discussion

We have developed a model of satellite-based environmental data-fishing performance relationship to explore and map out the spatial distribution pattern and persistence of pelagic hotspots for skipjack tuna. The fishing performance data represented by CPUE and fishing frequency are low-cost fish distribution datasets commonly available to fishery scientists. CPUE data provide a good proxy as an index of fish abundance [15,36], whereas fishing frequency data act as an index of fish occurrence or fish availability [7,37]. The fishing data describe fisher’s experience-based knowledge and provide invaluable supplement data to a better habitat prediction [38], while satellite data are mostly available at no cost to the user monitoring oceanographic features over a wide area [9,39]. Therefore, the strong correlation between the fishery data with the satellite oceanographic information provides an important means to identify habitat hotspots for pelagic species.

In principle, our model extracts the optimum combination of three environmental factors (SST, Chl-a and SSHA) from the areas of high fishing performance to produce pelagic habitat hotspots. Several studies supported that a combination of these factors plays a pivotal role in explaining and exposing a pelagic tuna habitat [7,8,22,40]. Our results found that Chl-a is an important variable for identifying tuna forage habitat [9]. SST was selected to be another important variable for detecting the habitat hotspot since skipjack tuna are sensitive to the changes of temperature on their distribution [15], while SSHA is related to the changes in the depth of the thermocline and mesoscale variability [39,41]. We combined these variables to improve detection of potential pelagic hotspots for skipjack tuna.

Our results show that skipjack tuna habitat is associated with the areas of warm SST, specific Chl-a concentrations and positive SSHA, favoring fishing operations (Figs 2 and 3). The surface temperature preference for skipjack is relatively warmer than reported from the other areas around the world [15,22,27,28,37]. The highest catches consistently occur in May when SST gradually decrease to about 30.5°C after reaching a peak in November-December and back to the lowest SST in July and August [34]. At the same time, the skipjack tuna fishery tends to occur within the areas of positive SSHA anomalies suggesting that food aggregates mainly at the surface when the thermocline depth moves in the opposite direction of sea surface height [41,42]. We found that predominantly positive SSHA had an effect on both skipjack CPUE and number of fishing set (Fig 3C), reflecting preference for areas closely associated with the warm mixed layer above the thermocline. The positive SSHA values are preferred for the skipjack tuna habitat [22,27], indicating the important anticyclonic eddy fields [43,44] where skipjack catches increase significantly [45].

It is interesting to note that our finding shows Chl-a as a key oceanographic indicator of locating hotspots for skipjack tuna within the southwestern Coral Triangle tuna. Satellite derived Chl-a concentration is an index of phytoplankton biomass which provides valuable information about trophic interactions, forage habitat and dynamic movement of pelagic species [9,46,47]. Skipjack feed on both the small epipelagic fish and zooplankton which all of them graze on phytoplankton [46]. The Chl-a concentrations control the skipjack abundance (CPUE) in the food web system through the linkages between phytoplankton and, zooplankton and small pelagic fish. Therefore skipjack tuna enable to take advantage of a short food-web which is probably efficient from the energetic point of view [48]. We show that favorable Chl-a for skipjack has more specific range than the previous study [22] and clearly indicates frontal areas at the level of 0.2 mg m^-3^ Chl-a isopleth (Fig 4). Skipjack tuna fishing sets assembled in waters along Chl-a front (Figs 2 and 4), implying that this oceanographic feature plays a role for detecting skipjack habitat hotspot along study area (Figs 5 and 8). Preference for 0.2 mg m^-3^ Chl-a has important biophysical, physiological and trophic implications. Skipjack tuna locate and forage along the frontal zones within the preferred temperatures and SSHAs [18,22,49].

In the present paper, we explore the performance of skipjack hotspots based on the three main points: (1) high PHI; (2) the area of potentially suitable habitat and (3) persistence of the most suitable habitat. For the period of 5 years, our findings show that the areas of the most potential skipjack habitat hotspot consistently peak in May corresponding to the highest PHI (Figs 5 and 6). These areas may relate strongly with enhanced feeding opportunities for skipjack. Several investigations found that the distribution and abundance of tuna are strongly linked with the forage availability [23,50,51]. Skipjack tuna move and exploit primarily high densities of food organisms, which could be tracked by the high PHI. The skipjack forage on species such as anchovy, cephalopods and crustacean [48], which are more abundant in the areas of increased probability index. Anchovy (*Stolephorus spp*.) represent more than 80% of skipjack stomach content when caught in the western Coral Triangle tuna [52]. We propose that the PHI provides a reasonable proxy detecting forage abundance, and thus skipjack tuna spatial distribution and abundance. The key skipjack habitats in the peak season (May) may have a good association with enhanced feeding opportunity for skipjack which is probably stimulated by the Chl-a front (Figs 4,8 and 10), upwelling zone [31,43], ocean current and eddy fields [43,44,53].

Although the formations of habitat hotspot varied spatially (Fig 5), however, the spatial mean position of the pelagic hotspots did not substantially change (Fig 10). Habitat hotspots in the Gulf of Bone such as in 2007 appear more pronounced than in the Flores Sea reflecting that the enhanced forage habitat supporting high tuna concentration cover a wide area. In the subsequent years, the biologically rich habitats mainly perform along the Chl-a of 0.2 mg m^-3^ (Chl-a front). Several explanations for the association of tunas with fronts include: (1) the availability of appropriate food; (2) confinement to a physiologically optimum temperature range; (3) use of frontal gradients for thermoregulation; (4) limitation of visual hunting efficiency owing to water clarity [49]; and (5) forage habitat and migration route [9,54]. The Chl-a front appears to coincide with the shelfbreak position (approximately 350 m isobath) of both Flores Sea and Bone Gulf (Figs 1,5 and 10). Highest skipjack CPUEs are concentrated near the shelfbreak location [17] during the daytime [55]. Our empirical fishing data (2007–2011) confirm that fishermen consistently exploit the forage habitat during the daytime and, using the fishing data in 2012, we showed that our prediction models have been substantially verified (Figs 5, 8 and 9). To improve the model performance, we suggest that the effect of upwelling and current systems should be added to the analysis.

It is important to note that at the peak season for 5-years (May 2007–2011), less than 2% of the study area exhibits a persistent concentration of habitat hotpots (Fig 10: left). We suggest that these areas play a pivotal role since skipjack CPUEs increase significantly with increasing the habitat persistence (Fig 10: right) and thereby provide potential targets for marine conservation and fishing management strategies. As a result, our findings could be as preliminary nature of results in providing new insight into detection of skipjack tuna distribution and abundance in either the Coral Triangle tuna region or the western tropical Pacific Ocean.

## Conclusions

Pelagic habitat hotspots for skipjack tuna in the southwestern Coral Triangle tuna are influenced by the optimum combination of environmental factors (SST, Chl-a and SSHA) detectable from satellite images. Skipjack CPUEs increased significantly in the areas of highest pelagic hotspot index (PHI). We found the key pelagic habitat corresponded mainly with the Chl-a front, which could stimulate enhanced forage abundance for skipjack within a physiologically optimum temperature range above the thermocline depth. The habitat hotspot and its persistence are clearly identified by 0.2 mg m^-3^ Chl-a isopleth, suggesting that the Chl-a front provides an important step on detection of habitat hotspots, distribution patterns and abundance of skipjack tuna in the western tropical Pacific Ocean, especially within Coral Triangle **t**una.

## Supporting information

S1 Fig The spatial distribution of SST for May 2007–2011 estimated from MODIS ocean color data. The dash lines correspond to the approximate optimum SST range.
(PDF)

S2 Fig The spatial distribution of SSHA for May 2007–2011 estimated from AVISO—altimetry. The dash lines indicate the approximate optimum SSHA range.
(PDF)
