# Supplementary figures and images for: Detection of pelagic habitat hotspots for skipjack tuna in the Gulf of Bone-Flores Sea, southwestern Coral Triangle tuna, Indonesia
Source: PLoS One. 2017 Oct 2;12(10):e0185601. doi: 10.1371/journal.pone.0185601 (PMC5624707; doi:10.1371/journal.pone.0185601)

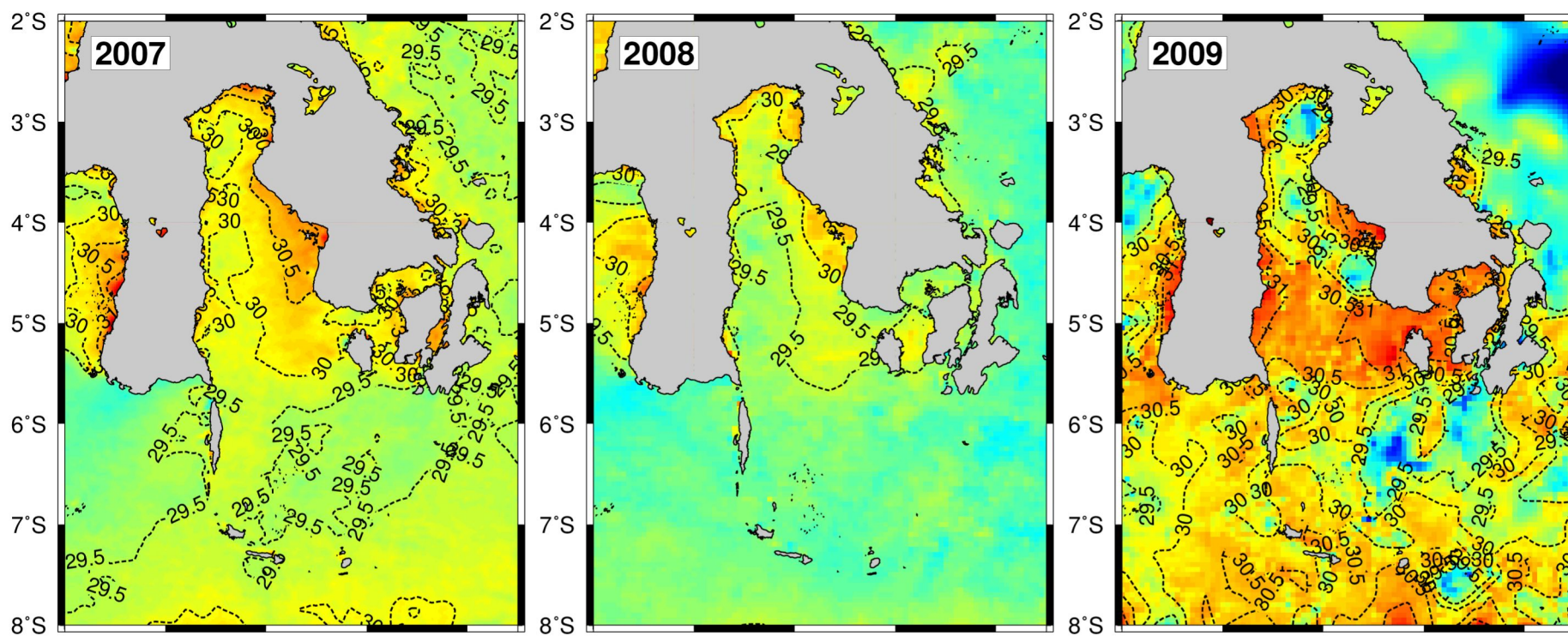

Supplement: S1 Fig — The dash lines correspond to the approximate optimum SST range. (PDF) [file pone.0185601.s001.pdf]

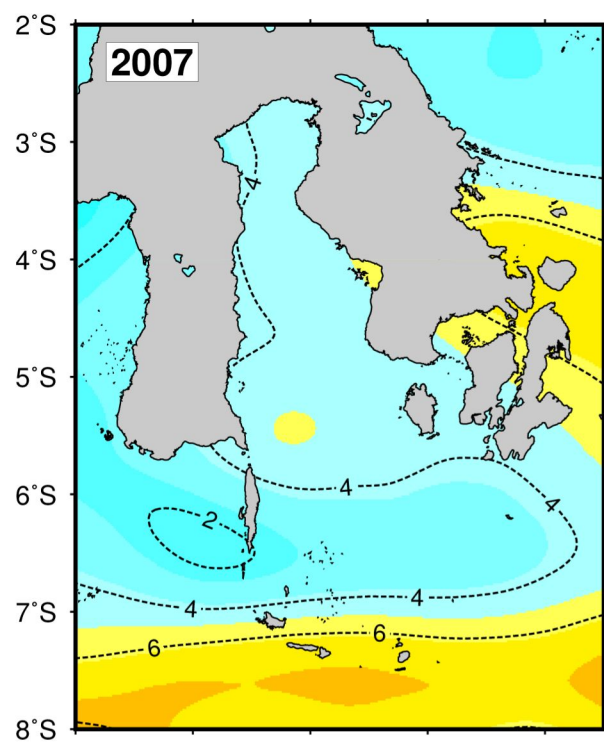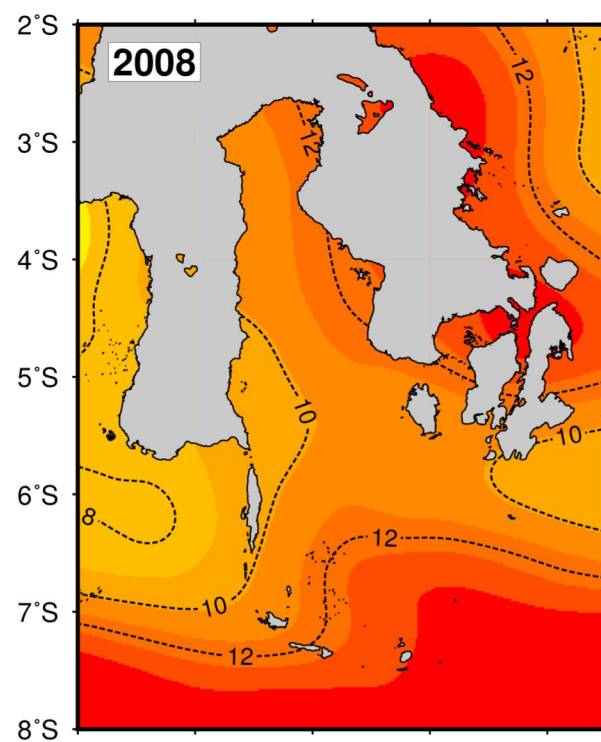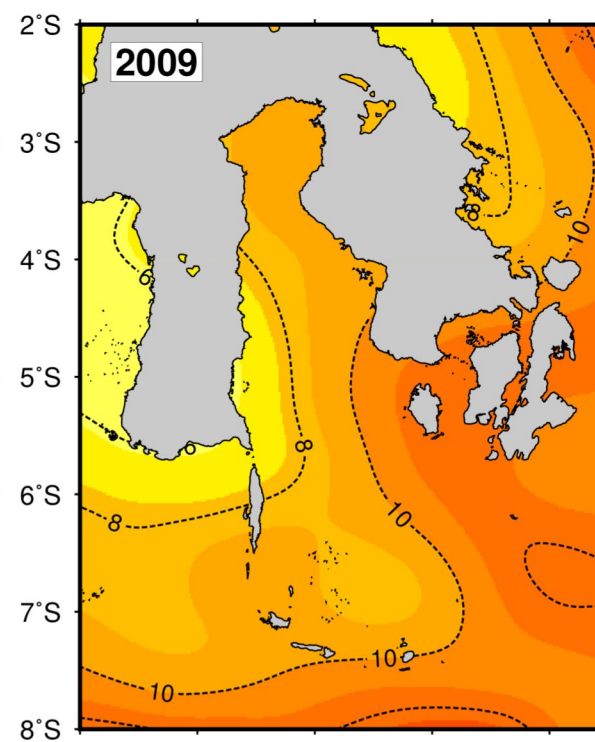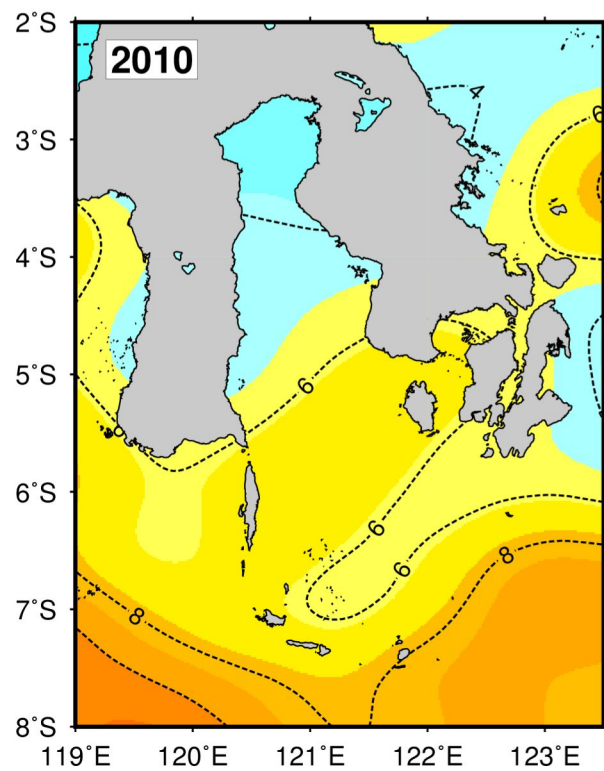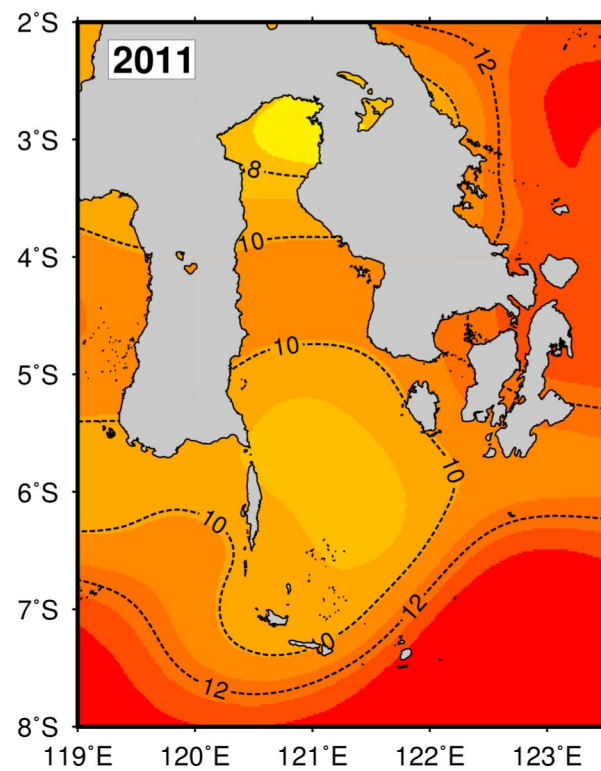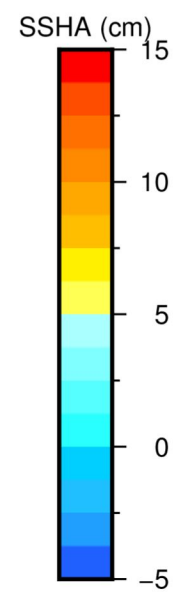

Supplement: S2 Fig — The dash lines indicate the approximate optimum SSHA range. (PDF) [file pone.0185601.s002.pdf]
